# Chromatin condensation of *Xist* genomic loci during oogenesis in mice

**DOI:** 10.1242/dev.127308

**Published:** 2015-12-01

**Authors:** Atsushi Fukuda, Atsushi Mitani, Toshiyuki Miyashita, Akihiro Umezawa, Hidenori Akutsu

**Affiliations:** 1 Center for Regenerative Medicine, National Research Institute for Child Health and Development, 2-10-1 Okura, Setagaya, Tokyo 157-8535, Japan; 2 Department of Molecular Genetics, Kitasato University Graduate School of Medical Sciences, 1-15-1 Kitasato, Minami, Sagamihara, Kanagawa 252-0374, Japan; 3 Department of Stem Cell Research, Fukushima Medical University, 1 Hikarigaoka, Fukushima City, Fukushima 960-1295, Japan

**Keywords:** *Xist*, Imprinted XCI, Chromatin condensation, Oogenesis, Histone methylation, Nuclear transfer

## Abstract

Repression of maternal *Xist* (Xm-*Xist*) during preimplantation in mouse embryos is essential for establishing imprinted X chromosome inactivation. Nuclear transplantation (NT) studies using nuclei derived from non-growing (ng) and full-grown (fg) oocytes have indicated that maternal-specific repressive modifications are imposed on Xm-*Xist* during oogenesis, as well as on autosomal imprinted genes. Recent studies have revealed that histone H3 lysine 9 trimethylation (H3K9me3) enrichments on Xm-*Xist* promoter regions are involved in silencing at the preimplantation stages. However, whether H3K9me3 is imposed on Xm-*Xist* during oogenesis is not known. Here, we dissected the chromatin states in ng and fg oocytes and early preimplantation stage embryos. Chromatin immunoprecipitation experiments against H3K9me3 revealed that there was no significant enrichment within the Xm-*Xist* region during oogenesis. However, NT embryos with ng nuclei (ngNT) showed extensive Xm-*Xist* derepression and H3K9me3 hypomethylation of the promoter region at the 4-cell stage, which corresponds to the onset of paternal *Xist* expression. We also found that the chromatin state at the *Xist* genomic locus became markedly condensed as oocyte growth proceeded. Although the condensed Xm-*Xist* genomic locus relaxed during early preimplantation phases, the extent of the relaxation across Xm-*Xist* loci derived from normally developed oocytes was significantly smaller than those of paternal-*Xist* and ngNT-*Xist* genomic loci. Furthermore, Xm-*Xist* from 2-cell metaphase nuclei became derepressed following NT. We propose that chromatin condensation is associated with imprinted *Xist* repression and that skipping of the condensation step by NT leads to *Xist* activation during the early preimplantation phase.

## Abstract (precis)

**Summary:** The analysis of chromatin state and H3K9me3 levels in mouse oocytes and early embryos provides insights into the dynamics of *Xist* repression and activation during early development and reprogramming.

## INTRODUCTION

Expression of the large non-coding RNA X inactive specific transcript (*Xist*) is essential for the initiation of X chromosome inactivation (XCI) in female mice and humans (Augui et al., 2011; Sado and Sakaguchi, 2013; Lee, 2011). In mice, *Xist* expression is initiated around the 4-cell stage and is restricted to the paternal allele (Augui et al., 2011; Sado and Sakaguchi, 2013; Nesterova et al., 2001). This expression pattern leads to the establishment of imprinted XCI in extra-embryonic tissues (Takagi and Sasaki, 1975). Paternal *Xist* (Xp-*Xist*) expression is driven by the deposition of maternal Rnf12 (also known as Rlim – Mouse Genome Informatics) (Shin et al., 2010; Jonkers et al., 2009). However, the *Xist* locus on the maternal X chromosome (Xm) is tightly protected by epigenetic factors. Using parthenogenetic embryos, which are composed of two maternal genomes, we previously demonstrated that histone 3 lysine 9 trimethylation (H3K9me3) is essential for Xm-*Xist* repression during early preimplantation phases (Fukuda et al., 2014).

Using a nuclear transplantation (NT) technique, bi-maternal embryos were constructed from non-growing (ng) and fully grown (fg) oocytes (Kono et al., 1996). XCI in the extra-embryonic tissues of bi-maternal embryos predominantly occurred on the allele from ng oocytes (Tada et al., 2000). The results indicated that the *Xist* loci of ng oocytes are in specifically permissive states for activation and that Xm-*Xist* imprints are established during oogenesis, as are those of autosomal imprinted genes. However, Xm-*Xist* silencing was observed in primordial germ cells (Sugimoto and Abe, 2007), suggesting that *in vivo*, repressive modifications were already imposed on the Xm-*Xist* prior to oogenesis. In addition, *Xist* dysregulation commonly occurred in cloned mouse embryos from various cell types such as somatic and embryonic stem cells (Inoue et al., 2010; Fukuda et al., 2010). Considering that NT is an artificial system, it does not exclude the possibility that NT embryos might not be faithfully reprogrammed. Therefore, in the present study we scrutinised the regulation of Xm-*Xist* by H3K9me3 and chromatin state in NT embryos derived from ng oocytes (ngNT).

## RESULTS AND DISCUSSION

### H3K9me3 is comparable between ng and fg oocytes at Xm-*Xist* loci

**Fig. 1.**
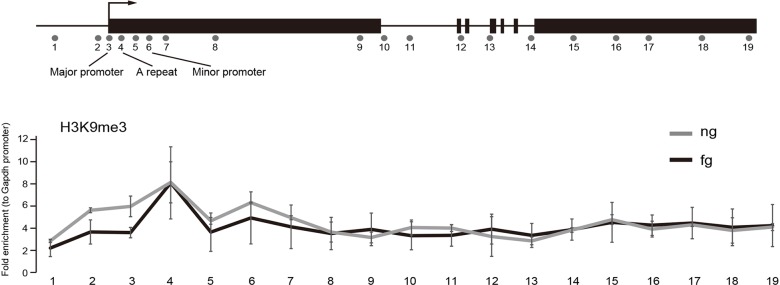
**H3K9me3 states in ng and fg oocytes by eChIP-qPCR analysis.** A total of 19 regions in *Xist* were analysed by eChIP-qPCR. Positions 3, 4, and 6 were localised in the major promoter, A repeat, and minor promoter, respectively. There were no significant differences among the regions tested. Three independent experiments were carried out, and the error bars show the standard error of the mean (s.e.m.).

We initially confirmed that Rnf12 is highly expressed during oogenesis (Fig. S1), as described elsewhere (Shin et al., 2010), indicating that the *Xist* repressive state is established prior to oocyte maturation. We previously demonstrated that H3K9me3 is essential for Xm-*Xist* repression in preimplantation embryos (Fukuda et al., 2014). To examine the chromatin states at *Xist* loci, we used an advanced system of embryo chromatin immunoprecipitation combined with TaqMan gene expression (eChIP-qPCR), which facilitated chromatin analysis of many loci from small numbers of cells. We targeted 19 regions in the *Xist* genes containing promoter regions and a *Gapdh* promoter region as a negative control region for H3K9me3 modification. Our eChIP-qPCR system robustly correlated with the conventional method (no preamplification) (1.21 to 1.57-fold increase in eChIP-qPCR, correlation between the two methods was >0.96; Fig. S2). We therefore examined H3K9me3 in ng and fg oocytes. eChIP-qPCR in ng oocytes revealed that the H3K9me3 levels of the 19 *Xist* regions examined were markedly higher than that of the *Gapdh* promoter region (Fig. 1); specifically, the levels in the *Xist* promoter regions were up to 6-fold higher. The repressive states across the entire *Xist* region were maintained in fg oocytes, and there were no significant differences between ng and fg oocytes in H3K9me3 levels at any of the *Xist* regions analysed (Fig. 1). These results indicated that transcriptional repressive states were imposed by H3K9me3 in ng oocytes and that the modifications were not established during oogenesis.

### Specific loss of H3K9me3 at Xm-*Xist* promoter regions following NT

Our findings showed that the *in vivo* repressive histone H3K9me3 modifications were already imposed on Xm-*Xist* prior to the initiation of oogenesis. Generally, immunofluorescence (IF) analysis showed that after fertilisation, global H3K9me3 was specifically imposed on the maternal genome (Santos et al., 2005; Kageyama et al., 2007). Interestingly, the lack of H3K9me3 was not restricted to the sperm genome. IF analysis revealed that global H3K9me3 levels at the 1-cell stage were markedly lower in the genomes from somatic and embryonic stem cells (ESCs) compared with maternal genomes (Wang et al., 2007) (Fig. S3A). However, the dramatically low H3K9me3 levels in ESCs and sperm genomes were not observed at the 2- and 4-cell stages (Fig. S3B). These observations implied that the relaxed chromatin state characterised by low H3K9me3 levels at the 1-cell stage might be important for subsequent *Xist* expression in early preimplantation phases.

**Fig. 2.**
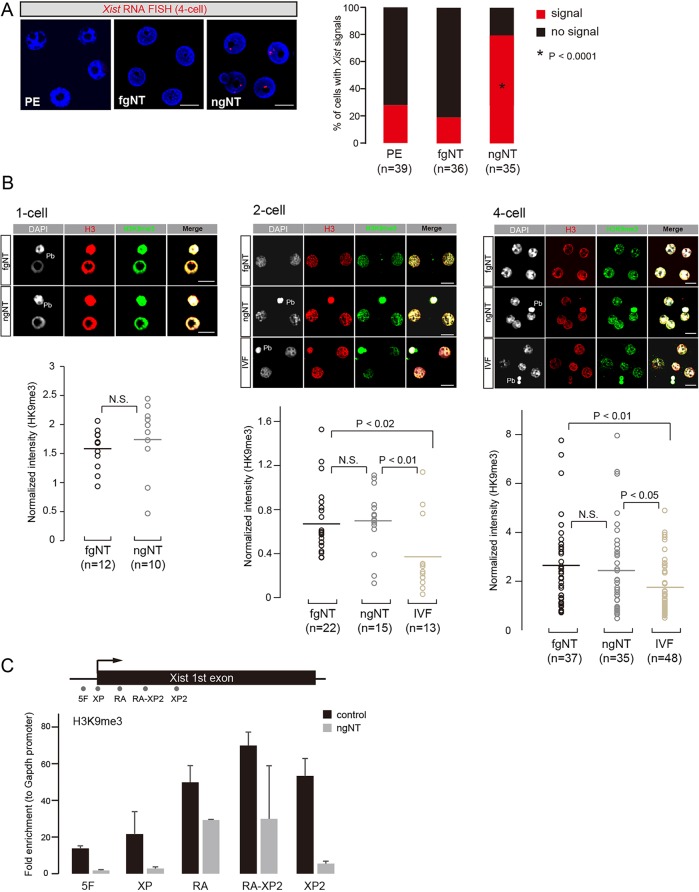
**Loss of H3K9me3 in ngNT embryos at Xm-*Xist* promoter regions but not genome wide.** (A) *Xist* RNA-FISH analysis at the 4-cell stage of ngNT (diploid ng genomes), fgNT (diploid fg genomes), and parthenogenetic (diploid fg genomes) embryos. Nuclei stained with 4′,6-diamidino-2-phenylindole (DAPI) are shown in blue. *Xist* is shown in red. *n*=number of analysed nuclei. The *P*-values were calculated using Fisher's exact test (compared with PE and fgNT, respectively). (B) IF analysis of H3K9me3 in ngNT embryos and control (fgNT) embryos constructed by serial NT at the 1-cell stage. fgNT and ngNT embryos were produced by single NT at 2- and 4-cell stages. For comparison with fertilised embryos, *in vitro* fertilised (IVF) embryos were prepared. *n*=number of analysed nuclei. Scale bar: 20 μm. DAPI, white; H3, red; H3K9me3, green. Pb, polar body. The H3K9me3 signal intensity was normalised by the H3 signal. *P*-values were calculated using Student's *t*-test. N.S., not significant. (C) eChIP-qPCR analysis of ngNT and control (tetraploid fgNT) embryos at the 4-cell stage. H3K9me3 at Xm-*Xist* promoter regions of tetraploid embryos in both groups were analysed by eChIP-qPCR. Two independent experiments were conducted, and error bars show s.e.m.

To ascertain chromatin states, we constructed NT embryos with ng oocyte genomes (ngNT) and performed IF analysis against H3K9me3. We first examined whether the Xm-*Xist* of ngNT was derepressed at the 4-cell stage. Fluorescence *in situ* hybridisation (FISH) analysis for *Xist* RNA showed extensive *Xist* expression (Fig. 2A), confirming that derepression of Xm-*Xist* in ngNT commenced at early preimplantation phases. Next, we conducted IF analysis for H3K9me3 at the 1-cell stage in ngNT constructed by serial NT (Kawahara et al., 2008). Unexpectedly, compared with control embryos (fgNT), there were no apparent reductions in H3K9me3 modifications in ngNT, and the same modifications were observed at the 1-cell to 4-cell stage (Fig. 2B). The signals of H3K9me3 in ngNT and fgNT were significantly higher than those of fertilised embryos (IVF, parental genomes; Fig. 2B), consistent with a previous report that Ring1b (also known as Rnf2 – Mouse Genome Informatics) – but not H3K9me3 – is enriched in paternal constitutive heterochromatin (Puschendorf et al., 2008).

We also examined the expression states of H3K9me3-associated genes (Peters et al., 2001; Matsui et al., 2010; Greer and Shi, 2012; Klose and Zhang, 2007) (erasers: *Kdm4a/b/c*, writers: *Suv39h1/2*, *Setdb1*) at the 4-cell stage of ngNT and found that the expression levels varied among the ngNT, as well as between control group embryos (Fig. S3C). Although only *Kdm4a* was markedly reduced in most ngNT, it did not seem to affect H3K9me3. These results indicated that global ng genomic H3K9me3 following NT was comparable to that of the fg oocyte genome.

However, a previous study has shown Xm-*Xist* activation to be accompanied by promoter demethylation (Fukuda et al., 2014). Thus, we next asked whether Xm-*Xist* derepression of ngNT embryos at the 4-cell stage could be attributed to the loss of H3K9me3 at *Xist* promoter regions. To reduce the number of embryos required for eChIP-qPCR analysis, we constructed tetraploid ngNT by repressing second polar body release. The tetraploid ngNT also showed Xm-*Xist* derepression at the 4-cell stage (Fig. S4). As expected, H3K9me3 modifications at *Xist* major promoter regions in tetraploid ngNTs at the 4-cell stage declined dramatically (less than 15% of the control) (Fig. 2C). The A repeat regions showed slight demethylation (around 60% of control), consistent with previous results of H3K9me3 demethylase-mediated Xm-*Xist* derepression (Fukuda et al., 2014). Thus, intrinsic Xm-*Xist* protection by H3K9me3 was not maintained following NT.

### Genome-wide loss of H3K9me2 in ngNT embryos at the 1-cell stage

We further examined H3K9me2 and H3K27me3 in ngNT embryos because both were shown to be specifically imposed on maternal genomes in zygotes (Fukuda et al., 2014; Santos et al., 2005; Nakamura et al., 2012). H3K27me3 levels of ng and fg oocyte genomes were comparable (Fig. S5A). However, H3K9me2 signals in ngNT were much lower than those in fgNT (Fig. S5A), although low levels of global H3K9me2 were only observed at the 1-cell stage, with no apparent differences at the 2- and 4-cell stages (Fig. S5B). Although the loss of H3K9me2 in the maternal genome did not affect Xm-*Xist* derepression (Fukuda et al., 2014), given that H3K9me2 is inversely related to gene expression at a genome-wide scale (Wen et al., 2009), the genome-wide lack of H3K9me2 at the 1-cell stage suggested that the chromatin of ng oocytes might be loosened following NT.

### Chromatin condensation of *Xist* genomic loci during oogenesis and relaxation in early preimplantation

**Fig. 3.**
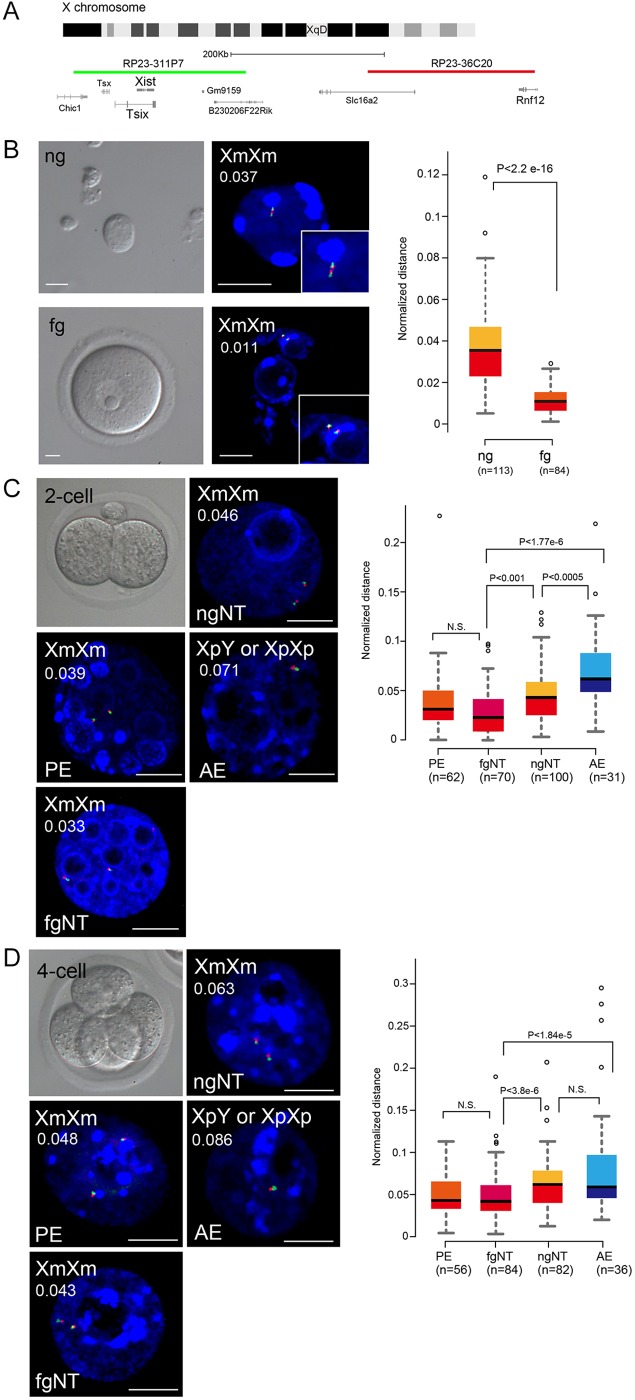
**DNA FISH analysis at the *Xist* genomic loci in ng and fg oocytes and various preimplantation embryos.** (A) Measurement of the distance between centroids of each signal by DNA-FISH using BAC clones. The BAC clone colours, green for RP23-311P7 and red for RP23-36C20, correspond with the signals in B-D. (B-D) DNA-FISH analysis in ng and fg oocytes (B) and in parthenogenetic (PE: diploid fg genomes), fgNT (diploid fg genome), ngNT (diploid ng genomes), and androgenetic (AE: diploid sperm genomes) embryos at 2-cell (C) and 4-cell (D) stages. *n*=number of analysed signals. The boxplot indicates the normalised distances, and *P*-values were calculated by Mann–Whitney *U*-tests. Xm, maternal X chromosome; Xp, paternal X chromosome. Scale bar: 10 μm. Nuclei (DAPI), blue. Average values of normalised distance are given top left in each image.

Consistent with the above notion, DNA methylation levels were shown to dramatically change during oogenesis, and high levels of DNA methylation were observed only in fg oocyte genomes (Shirane et al., 2013). We speculated that maternal genomes might become transcriptionally silent via chromatin condensation. To test this, we carried out DNA-FISH experiments using probes spanning XqD, which contains *Xist*, and measured the distance between loci (Fig. 3A). Many studies have normalised distance with this method by calculating the nuclear radius visualised by 4′,6-diamidino-2-phenylindole (DAPI) staining (Terranova et al., 2008; Eskeland et al., 2010; Chambeyron and Bickmore, 2004). However, the chromatin in fg oocytes surrounds the nucleolus, and the DAPI-positive area does not totally cover the regions enclosed by the nuclear membrane (De La Fuente, 2006). Therefore, to determine accurate nuclear radii in fg oocytes, we conducted IF against histone deacetylase 2 (Hdac2), which occurs specifically in the nuclear area. Furthermore, a report has shown the predominant expression of Hdac2 in fg oocytes during oogenesis (Fig. S6A) (Ma et al., 2012). The distance in the fg oocyte genome was therefore normalised by the average radius, whereas the distance of the ng oocyte genome was normalised by the DAPI-positive nuclear area (average of ng oocyte examined). DNA-FISH analysis showed that the genomic regions containing *Xist* were significantly condensed in fg oocytes even without normalisation (Fig. 3B, Fig. S6B), indicating that the chromatin of the *Xist* genomic locus became condensed during oogenesis.

Next, we conducted DNA-FISH experiments at the 2- and 4-cell stages in ngNT, parthenogenetic (derived from fg oocytes), and androgenetic embryos (containing *Xist* genomic loci derived from paternal X chromosomes). At the 2-cell stage, the distance between loci in ngNT embryos was significantly larger than that of parthenogenetic and fgNT embryos, but smaller than that of androgenetic embryos (Fig. 3C). However, at the 4-cell stage, the distance was comparable to that of androgenetic embryos but significantly larger than that of parthenogenetic and fgNT embryos (Fig. 3D). Thus, the open chromatin state in ng oocytes was maintained following NT, suggesting that chromatin condensation is likely essential for *Xist* repression.

### Imprinted Xm-*Xist* silencing in early embryonic cells is not reprogrammed following NT

**Fig. 4.**
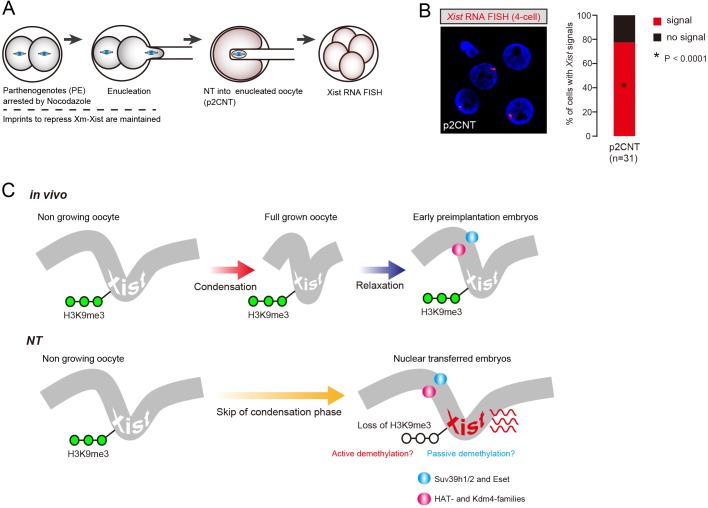
**Imprinted Xm-*Xist* silencing in early embryonic nuclei are not reprogrammed following NT.** (A) Experimental scheme of embryonic NT. Diploid parthenogenetic 2-cell embryos were incubated in the presence of Nocodazol (0.1 μg/ml) for 12 h and washed in M2 medium to form spindles. Condensed nuclei of metaphase-arrested 2-cell parthenogenotes (PE) were transferred into enucleated oocytes (p2CNTs). (B) *Xist* RNA-FISH analysis of p2CNT embryos at the 4-cell stage. The bar graph shows the *Xist* expression states in each embryo. *P*-values were calculated by Fisher's exact tests in comparison with the PE and fgNT embryos in Fig. 2A. (C) Model of Xm-*Xist* silencing machinery from oogenesis to early preimplantation phases. During oogenesis, Xm-*Xist* loci are condensed, but become relaxed in early preimplantation phases. NT skips the condensation phase to result in Xm-*Xist*, which is in a permissive state for activation. During oocyte and early preimplantation stages, some H3K9me3 demethylases and histone acetylases are expressed (Fukuda et al., 2014; Yoshida et al., 2007). Open chromatin might cause active or passive demethylation.

We also found that the distance between *Xist* genomic loci became larger following cell division (Fig. 3B-D), implying that chromatin becomes gradually relaxed during the early cleavage stage, probably reflecting zygotic gene activation. Thus, we hypothesised that although 4-cell stage parthenogenetic embryo Xm-*Xist* was silenced, the chromatin might be looser than in fg oocytes and thus might be derepressed following NT. We therefore produced 2-cell parthenogenotes arrested at metaphase by Nocodazol treatment and conducted NT experiments (Egli et al., 2009) (Fig. 4A). *Xist* RNA-FISH revealed that Xm-*Xist* of NT embryos derived from arrested nuclei of 2-cell parthenogenotes (wherein imprinted *Xist* silencing was maintained) was robustly expressed at the 4-cell stage (77% of nuclei; Fig. 4B). These results indicated that imprinted *Xist* repression associated with open chromatin states in donor cells was not faithfully reprogrammed following NT.

### Conclusions

As imprinted *Xist* expression is not common in other species, the observed genome condensation during oogenesis might specifically occur on the murine X chromosome. Accordingly, we found that X-linked gene expression levels in mice markedly declined during oogenesis, whereas they were only slightly reduced in mature human oocytes (Fukuda et al., 2015).

NT studies in mice showed *Xist* upregulation regardless of donor cell origins (Fukuda et al., 2010), even if the *Xist* imprint was maintained in donor cells such as from early preimplantation embryos (Fig. 4B). Furthermore, considering that H3K9me3 demethylases and histone acetyltransferases are expressed in oocytes (Fukuda et al., 2014; Yoshida et al., 2007), the Xm-*Xist* promoter in ngNT could be subjected to demethylation owing to chromatin decondensation (Fig. 4C). Overall, our results suggest – as previously proposed by Sado and Sakaguchi (2013) – that chromatin condensation is associated with imprinted *Xist* repression on the maternal X at the early preimplantation stage, and that skipping of the condensation step by NT leads to precocious activation of *Xist* in early preimplantation embryos.

## MATERIALS AND METHODS

### Animals

All mice were maintained and used in accordance with the Guidelines for the Care and Use of Laboratory Animals of the Japanese Association for Laboratory Animal Science and the National Research Institute for Child Health and Development of Japan (A2006-009-C09).

### Oocyte and sperm collection

Female B6D2F1 and C57BL/6N mice were purchased from Clea Japan, and oocytes were collected following standard methods. The ng and fg oocytes (C57BL/6N) were recovered from new-born (1- to 5-day-old) and adult (8- to 12-week-old) mice according to previous reports (Kawahara et al., 2008). For eChIP-qPCR analysis, ng oocytes were collected using a micromanipulator. Sperm was collected from male B6D2F1 (8-16 weeks) mice according to reported methods (Fukuda et al., 2014).

### Embryo manipulations

Parthenogenetic embryos were generated using calcium-free M16 medium containing 8 mM SrCl_2_ and 5 μg/ml cytochalasin B (Sigma-Aldrich) (activation medium) and were cultured in KSOM (EMD Millipore). Serial NT of ng oocytes for immunofluorescence analysis was conducted as described (Kawahara et al., 2008). Single NT for ng oocyte nuclei was conducted using a Piezo drive (Sutter Instrument Company) or hemagglutinating virus of Japan envelope (HVJ-E; Ishihara Sangyo Kaisha) (Kawahara et al., 2008; Egli et al., 2009). To produce diploid ngNT embryos for DNA FISH analysis, cytochalasin B was removed from the activation medium, whereas tetraploid ngNT embryos were generated by adding cytochalasin B to the activation medium. For construction of tetraploid fgNT, an MII oocyte received a nucleus from another MII oocyte by NT and then the NT embryo was parthenogenetically activated in the presence of cytochalasin B. The diploid androgenetic embryos were generated using a previously reported method (Kono et al., 1993). For ESC injection, ovulated MII oocytes at 12-13 h after human chorionic gonadotropin (hCG) injection were recovered to retain the first polar body, and ESCs were injected using the Piezo drive. All embryos were cultured at 37°C in KSOM in an atmosphere containing 5% CO_2_.

### Fluorescence *in situ* hybridisation (FISH)

RNA-FISH analysis was performed according to a previous report (Fukuda et al., 2014). In brief, an *Xist* probe (provided by T. Sado) was prepared using a Nick Translation Kit (Abbott Laboratories) and Cy3-dUTP (GE Healthcare Life Sciences).

For DNA-FISH, BAC clones (RP23-311P7: *Xist*/*Tsix* regions and RP23-36C20: *Slc16a2/Rnf12* regions) were purchased from Life Technologies. DNA probes of RP23-311P7 and RP23-36C20 were prepared using the Nick Translation Kit with Cy5-dUTP and Cy3-dUTP, respectively. The procedures were as previously reported. In brief, fixation (2% paraformaldehyde) and permeabilisation (0.25% Triton X-100) were simultaneously conducted for 5 min at room temperature, and then the samples were plated onto glass slides. After RNaseA treatment, the samples were incubated in 0.2 N HCl containing 0.5% Triton X-100 on ice for 10 min. The images were obtained by LSM510 laser scanning confocal microscopy using a Plan-Apochromat 100×/1.46 Oil DIC objective (Carl Zeiss).

Distance measurements were based on previous reports (Terranova et al., 2008; Eskeland et al., 2010; Chambeyron and Bickmore, 2004). Briefly, the signal centroid was calculated by NIH ImageJ software (http://rsb.info.nih.gov/ij/). Each nuclear radius, except for those of fg oocytes, used for distance normalisation was calculated using the DAPI-stained area measurement.

### eChIP-qPCR for oocytes and embryos

We prepared 300 fg and ng oocytes and 50 tetraploid 4-cell embryos for the eChIP-qPCR assay. Zona pellucida-free fg oocytes, embryos and ng oocytes were suspended in PBS containing 0.5% Triton-X100, 0.5 mM DTT and protease inhibitor (PBS + lysis buffer) and incubated on ice for 30 min. The chromatin was incubated with 100 Gel U/μl micrococcal nuclease (final 0.33 Gel U) (New England BioLabs) for 5 min at 37°C, after which the chromatin was extracted by centrifugation. After recovery of the supernatant, PBS + lysis buffer was added to the (invisible) pellet, and an additional treatment with 2000 Gel U/μl micrococcal nuclease (final 6.7 Gel U) for 5 min at 37°C was performed. The chromatin was then incubated with an antibody against H3K9me3 (Abcam, ab8898) conjugated with protein A (Dynabeads) overnight at 4°C. The pelleted beads were washed twice with both Buffer 1 (50 mM Tris-HCl pH 7.5, 500 mM NaCl, 10 mM EDTA) and Buffer 2 (50 mM Tris-HCl pH 7.5, 300 mM NaCl, 10 mM EDTA). The pelleted beads were suspended in ChIP direct elution buffer (Nippon Gene) and incubated with proteinase K for 2 h at 37°C (Roche). The immunoprecipitated DNA was then purified using Agencourt AMpure XP beads (Beckman). The DNA was preamplified using the preamplification mix from the Single Cell-to-CT Kit (Ambion) and TaqMan probes according to the manufacturer’s instructions (except that the number of PCR cycles was changed to 20). The primers/probes used are listed in Table S1.

### Immunofluorescence

The antibodies for H3K9me2 (Abcam, ab1220), H3K9me3 (Abcam, ab8898), H3K27me3 (Millipore, 07-449), H3 (Active Motif, 39763) and Hdac2 (Millipore, 05-814) were used for immunofluorescence (IF) analysis. For IF against histone methylation, fixation by 2% paraformaldehyde and permeabilisation with 0.25% Triton X-100 were simultaneously conducted. Fixation was followed by permeabilisation for Hdac2 IF analysis. After fixation and permeabilisation the embryos were blocked in PBS containing 1% BSA (blocking buffer) for 1 h and incubated with antibodies overnight at 4°C. The first antibodies for H3K9me2 (1:500) and H3K27me3 (1:300), or H3 (1:500) and H3K9me3 (1:500), were simultaneously incubated in blocking buffer. Embryos were washed with PBS containing 0.1% polyvinyl alcohol (PBS-PVA) and then incubated for 1 h at room temperature with Alexa Fluor 488- or 546-conjugated anti-mouse or anti-rabbit IgG secondary antibodies (Life Technologies; 1:500). After the embryos were washed with PBS-PVA, the nuclei were stained with DAPI. The zona pellucida was removed prior to fixation and permeabilisation (Fig. 2B). After the second antibodies were washed, the samples were attached onto slide glasses and observed.

### qPCR analysis of 4-cell stage embryos

The qPCR analysis was conducted using TaqMan probes (Life Technologies). For further details, see the supplementary Materials and Methods and Table S2.

### ESC culture

The culture of ESCs used for NT was according to a previous report (Fukuda et al., 2014). For further details, see the supplementary Materials and Methods.
